# Preservation and phylogeny of Cambrian ecdysozoans tested by experimental decay of *Priapulus*

**DOI:** 10.1038/srep32817

**Published:** 2016-09-06

**Authors:** Robert S. Sansom

**Affiliations:** 1 School of Earth and Environmental Sciences, University of Manchester, Manchester M13 9PT, UK

## Abstract

The exceptionally preserved Cambrian fossil record provides unique insight into the early evolutionary history of animals. Understanding of the mechanisms of exceptional soft tissue preservation frames all interpretations of the fauna and its evolutionary significance. This is especially true for recent interpretations of preserved nervous tissues in fossil ecdysozoans. However, models of soft tissue preservation lack empirical support from actualistic studies. Here experimental decay of the priapulid *Priapulus* reveal consistent bias towards rapid loss of internal non-cuticular anatomy compared with recalcitrant cuticular anatomy. This is consistent with models of Burgess Shale-type preservation and indicates that internal tissues are unlikely to be preserved with fidelity if organically preserved. This pattern, along with extreme body margin distortion, is consistent with onychophoran decay, and is therefore resolved as general for early ecdysozoans. Application of these patterns to phylogenetic data finds scalidophoran taxa to be very sensitive to taphonomically informed character coding, but not panarthropodan taxa. Priapulid decay also have unexpected relevance for interpretation of myomeres in fossil chordates. The decay data presented serve not only as a test of models of preservation but also a framework with which to interpret ecdysozoan fossil anatomies, and the subsequent evolutionary inferences drawn from them.

The Cambrian radiation of metazoan phyla is a fundamental and singular event in evolution of life on earth. The fossil record has been vital in interpreting the nature and timing of the event, as well as the circumstances that led to the construction of modern body plans and ecosystems, and by extension, the very nature of evolutionary processes. There are, however, many problems associated with the interpretation of the metazoan fossil record in the Cambrian, not only in terms of the evolutionary significance of the fauna, but also the mechanisms underlying its preservation.

Where we have a relatively complete picture of Cambrian ecosystems, it seems that the fauna were dominated by ecdysozoans^1^,^2^ and in particular, priapulids^3^. The Priapulida, or penis worms, played a key ecosystem role as predators and infaunal bioturbators. Priapulids are known from 7 modern genera, and a diverse Cambrian fauna^4^,^5^,^6^. Priapulids possess a tri-layered moulting cuticle diagnosing them as ecdysozoans, and a scalid lined introvert which they share with other scalidophorans (kinorhynchs and loriciferans)^7^. There is some ambiguity as to whether the Scalidophora are sister taxa to all other Ecdysozoa or are united with nematoids in the Cycloneuralia (Fig. 1)^8^,^9^,^10^,^11^,^12^. Either way, the priapulids are widely considered as representative of the morphological condition of the early ecdysozoans and are closest available proxy for the primitive condition for the group^13^. As such, they are the best model system for experimental investigation of mechanisms of ecdysozoan preservation and interpretations of their fossil record. Here the decay and preservation of *Priapulus caudatus* is experimentally investigated to address questions relating to interpretation of ecdysozoan fossil preservation and phylogeny. In particular, what are mechanisms by which the soft tissues are preserved and how do those mechanisms affect our ability to reconstruct relationships and assess evolutionary significance?

## Preservation

Multiple recent advances have been made with respect to understanding mechanisms of “Burgess Shale-type preservation” of soft tissues. A model has been proposed which posits that varying post preservational diagenesis belies much of the taphonomic variation observed between locations^14^,^15^. This builds on earlier interpretations that the primary type of soft tissue preserved in animals is cuticle and that other types of soft tissue are unlikely to be preserved^16^,^17^. Two main routes of preservation of soft tissues occur in Burgess Shale-type deposits: 1) organic preservation due to delayed decay of recalcitrant macromolecules such as collagen or chitin, and 2) rapid authigenic mineralization, for example bacterially mediated phosphatization^18^. These two pathways have very different timescales and capture very different kinds of information. Knowledge of taphonomy and timescale of decay is therefore fundamental to the interpretations of fossil morphology, and subsequently the evolutionary inferences drawn from them. This has been relevant to interpretations of controversial taxa such as *Pikaia* and *Amiskwia*^16^,^19^, but is also relevant to more recent interpretations of supposedly non-cuticular tissue in ecdysozoans. Nervous and vascular tissues have been described in Cambrian ecdysozoans, the morphology of which has been used to interpret phylogenetic relationships and evolutionary scenarios^20^,^21^,^22^,^23^,^24^,^25^. These fossil tissues have been demonstrated to have a similar style of preservation to other organically preserved recalcitrant morphology i.e. carbonaceous films with varying degrees of subsequent diagenesis (pyritization in the case of the Chengjiang biota^26^ or reflective films of the Burgess Shale^25^). As such, their interpretations as nervous or vascular tissues represents a “challenge [to] the widely accepted premise that authigenic mineralization is essential for the early fixation of labile tissues”^26^. In order to assess feasibility of preservation of these tissue types, empirical data about the timeframe of their decay and degradation is necessary. Following current models of preservation^18^, tissues found to be recalcitrant and slow to decay are generally expected to be organically preserved when found in fossils. Tissues found to be labile and rapid to decay should only be preserved under conditions that enable rapid authigenic bacterially mediated mineralization and should exhibit taphonomic properties that reflect that preservation mode (unless they leave behind pigmentation). Experimental decay can be extremely informative in this context, especially considering the unanticipated outcomes that can result^27^. Edgecombe *et al*.^28^ interpreted the supposed recalcitrance of the ventral nerve cord in the annelid *Nereis* as support for the feasibility of preservation of arthropod nervous tissue. The interpretation of those results is, however, problematic, given the requirements for hypotheses, design, and application of decay experiments^27^. Specimens of *Nereis* were encased in clay powder and stored at 7 degrees Celsius and dried for 1.5–2.5 months, then transferred to room temperature for 30–40 days before sampling. Dehydration, however, would be expected to have stopped decay altogether. They stated that “the durability of neural tissue in sediment suggests that decay experiments designed to exclude sediment from the equation are probably ignoring a vital factor in exceptional fossil preservation”^28^. However, the role of sediments was not experimentally assessed as a variable (e.g. comparing trials with and without sediments) so their role cannot be inferred. Furthermore, the recalcitrance of the tissues is reported in absolute terms (rather than relative to other tissues) and only for a short period in cool temperatures. Murdock *et al*.^29^ on the other hand, found nervous tissues to be highly labile relative to all other tissues in their experimental investigation of onychophoran decay. Onychophora make a better comparison for the Cambrian fossils in question given that they are ecdysozoans rather than lophotrochozoans. It is unclear, however, whether the results of terrestrial onychophorans can be applied more generally to the marine Cambrian fauna, or the role that sediments might have played, or whether the patterns are general for ecdysozoans. There are a wide range of possible variables that need to be taken into account for experiments investigating decay^27^. They can be loosely classified as organismal variables (the subject, its age and history) and environmental variables (physical, chemical and biological), one of which is the presence or type of sediment surrounding the decaying subject. The interactions between sediments and their subject (along with the microbial fauna) have been interpreted to play an important role in preservation and this has been experimentally demonstrated^30^,^31^. For example, Wilson and Butterfield^30^ found an interaction between sediment type and rates of decay in *Nereis* but not *Crangon*. Given the practical difficulties raised with the use of sediments^27^,^30^ as well as the large variability in them, many decay experiments use artificial seawater as a standard medium from which more complex models can be investigated^27^,^29^,^31^,^32^,^33^,^34^,^35^,^36^,^37^. Indeed whilst many variables are found to affect the rate of decay, sequence and patterns of decay appear to be consistent under many physical conditions^27^,^31^,^36^. For example, higher temperatures can cause a higher rate of decay, but the order of relative loss of anatomical characters during decay remains the same.

Here the relative decay susceptibility of internal and external characters of Priapulida is experimentally investigated, both with and without sediments. This not only serves as a test of the feasibility of non-cuticular tissue preservation, but in combination with results from decay of onychophorans, it enables proposal of a general model of decay and preservation for early ecdysozoans.

## Phylogeny

Understanding the processes of decay and fossil preservation can also have a direct impact on our interpretation of phylogeny and the evolutionary inferences drawn^38^. In the case of the soft-bodied organisms, including those from Burgess Shale-type deposits, knowledge of decay patterns is essential for interpretation of fossil anatomy. Whilst taphonomy may have been taken into account when interpreting taxa for previous phylogenetic analyses, such interpretations are rarely ever explicit, or available for scrutiny. Application of empirical decay data meanwhile enables the distinction to be made between absent characters that may have been lost to taphonomic processes (i.e. missing) and absent characters were never there in the first place (i.e. phylogenetically absent) as well as recognition of present but partially decayed characters^27^,^39^,^40^,^41^. Indeed the thresholds at which these sorts of coding errors create a significant problem for accurate phylogenetic inference are very low, thus underscoring the need for taphonomically informed coding approaches^35^,^40^,^42^. Furthermore, systematic loss of characters during preservation can systematically distort phylogenetic inferences^35^,^36^,^38^,^40^. In the case of chordates, experimental decay of soft tissues has revealed biases in the order of loss of characters which undermines fossil interpretation i.e. synapomorphic characters are lost early leaving plesiomorphic remains which may be interpreted as artifactually primitive unless decay processes are taken into account^35^,^36^. It is not clear how widespread this pattern of stem-ward slippage is across the tree of life. Although consistent for chordates^35^,^36^, it is not observed in onychophorans^29^ and enteropneusts^37^. Given the dramatic impact it could have on our understanding of the fossil record it is necessary to test for empirical decay biases for clades across the tree of life. Here the possibility that decay biases exist in Priapulida (and thus early ecdysozoans) is tested. Furthermore, the observed empirical patterns of character loss are applied to phylogenetic data to assess the effect of taphonomy on phylogeny.

## Materials and Methods

### Decay

*Priapulus caudatus* were collected during 5 days of benthos trawling at the Gullmar fjord, Sweden. They were stored in circulating deep fjord water (7 degrees Celsius) and sediment at the Sven Lovén Centre for Marine Sciences, Kristineberg for up to 10 days (January 2015). 19 individuals (ranging from 40–70 mm in length as well as one large individual at 120 mm length) were killed using an overdose of MS-222 (2 g/l ethyl 3-aminobenzoate methanesulfonic acid Sigma-Aldrich, with 4 g/l sodium bicarbonate buffer). MS-222 can have an adverse effect on endogenous bacterial communities, but this is mitigated by the use of a buffer^43^ (as applied here). Experimental procedures were adapted from Sansom *et al*.^35^,^36^,^41^ to accommodate the smaller number of individuals obtained (further sampling was deemed to have a prohibitively high ecological cost). Specimens were placed in individual polystyrene boxes (all 58 × 38 × 22 mm except one at 79 × 47 × 22 mm) filled completely with deep water extracted from the fjord (salinity 32–33 PSU, pH 8) that was first warmed slowly to room temperature. As such, the natural *in situ* microbial flora of the marine environment was present in the experimental vessels, alongside the flora endogenous to the subjects. Containers were closed with push fit lids sealed with silicone grease to limit oxygen diffusion. Plastic mesh floors (2 mm diameter holes) were added to 10 to facilitate extraction. To test for the effects of burial and sediment on decay, two individuals were placed in containers with natural sediments extracted from the fjord bottom (sifted at 0.5 mm and then 200 μm to extract macro-scavengers, then left to settle). For sediment trials, specimens were placed on plastic mesh with sediment filling the container both above and below (one additional container was filled with sediment and a plastic mesh but left otherwise barren). Specimens were placed on a hot plate (30 degree Celsius). For the next 6 days, they were photographed from above at a rate of once per hour (time-lapse), and one individual was terminally sampled on each day by photography, light microscopy, and dissection. On day 6 of decay, two specimens were sampled and the remaining were placed in an incubator (30 degree Celsius) and stored for 7.5 months (224 days). After this longer incubation, 5 specimens were sampled (three standard conditions and the two with sediment). Following Sansom *et al*.^35^,^36^, observable anatomical characters were identified and classified according to the hierarchical rank at which they are synapomorphic (Fig. 2). Characters synapomorphic for Ecdysozoa or Bilateria were grouped as ‘plesiomorphic’. As a phylum, Priapulida are characterized by an invaginable proboscis (introvert) bearing multiple longitudinal rows of scalids and pentagons of cuspidate teeth^6^,^44^. Some other characters that are general for Priapulida vary within the group and were treated as plesiomorphic (i.e. marked with an asterisk in Fig. 2)^5^,^6^. Characters were also classified as cuticular or non-cuticular (roughly equating to external and internal respectively). It was not possible to directly observe the internal neural tissue of the ventral nerve cord, only the corresponding groove in the external cuticular surface. It was therefore treated as a cuticular character. At each sampling interval, all characters for each specimen were scored and then ranked following Sansom *et al*.^35^,^36^ i.e. pristine (same morphology as fresh), decaying (altered morphology) or lost (no longer recognizable). The correlation of character decay rank against character synapomorphic rank was then calculated (Spearman’s rank correlation). The percentage completeness of characters was also used to test for differences between decay profile of cuticular and non-cuticular characters and specimens decayed with and without sediments (Mann-Whitney test).

### Phylogeny

To understand the impact that taphonomic loss of characters has upon phylogenetic reconstruction, trials were run on phylogenetic datasets for Priapulida^5^ and Panarthropoda^24^. Characters in each were classified as either cuticular or non-cuticular (the later being subdivided into non-cuticular anatomy, body proportions or developmental characters). The taxa were also classified as either extant or extinct. Fossil taxa were subdivided according to their general mode of preservation i.e. organic preservation or some other pathway (e.g. Orsten style preservation or preserved in amber). Trees resulting from searches with the complete original matrix were compared with trees resulting from searches with codings of organically preserved fossil taxa altered to reflect taphonomic patterns observed for Priapulida and Onychophora^29^ (i.e. characters unlikely to be preserved changed to missing). Taphonomic removal of character codings was compared against random removal of character codings in the same proportion in terms of the homoplasy of the resulting most parsimonious trees (retention index) and resolution of the strict consensus (number of nodes recovered). As such, the phylogenetic experiments serve to test the role of potentially dubious fossil codings in phylogeny, and the effects of their exclusion. Scripts were adapted from previous analyses^38^,^40^, and run using TNT^45^. Datasets were edited to remove parsimony uninformative characters. Tree searches were conducted with and without implied weighting following the original authors^5^,^24^.

A second test was conducted on the extant taxa to investigate the possible role of stem-ward slippage following taphonomic loss of characters. The original taxon positions were measured using taxon height (number of intervening nodes from tree root, averaged for all most parsimonious trees)^38^,^40^. These act as baselines to compare the relative shift of each extant taxon in turn under either systematic character removal (replacing codings for taphonomically unlikely characters with missing entries) and random character removal in the same proportion. As such, the experiment serves to test whether taphonomic removal of character codings results in consistent shifts in taxon positions outside the range expected given random removal of character codings^40^.

## Results

In the early stages of decay, within hours of death, the body of *Priapulus* undergoes extreme distortion in terms of both size and proportions (Fig. 3, supplementary movies). The sequence is loosely characterized as expansion, curvature, and contraction/relaxation. Non-uniform changes can result in irregular taphonomic bulging of the body. Alongside these changes, and potentially related to them, separation of the external and internal layers of cuticle occurs, and thus rapid formation of a large cavity between the two: the interior of the organism shrinks whilst the body margin expands (Fig. 4). Much of the shape changes appear to be related to extreme contraction of the longitudinal muscles. The interior non-cuticular anatomy undergoes markedly rapid decay. For example, the circumpharyngeal brain and introvert retractor muscles are lost completely after 4 days of decay (Fig. 2). The circular muscles become rapidly distorted changing shape from homonomous annulations to sigmoidal or double sigmoidal (W-shaped) when viewed laterally (Fig. 5). The external cuticular surface undergoes some decay (e.g. fraying of the external surface, base remaining generally intact) but it is generally very resistant to decay and undergoes little change during the course of the experiments (Fig. 6). The difference between the decay profile of non-cuticular and cuticle characters is highly significant (Mann-Whitney-Wilcoxon p = 0.00018). At the late stages of decay, the internal anatomy has decayed away and the external cuticle of the body remains generally intact and articulated. At this stage, the remains resemble those of an ecdysis moult, both in terms of anatomy and structural strength (Fig. 6). Within cuticular characters, the caudal appendage and trunk exhibited some decay whilst the majority (including all mouth scalid characters) underwent no decay at all over the timeframe of the experiment (7.5 months).

With respect to the specimens buried in sediment, the same general pattern was seen as the specimens decayed in water i.e. shrinkage and loss of the internal anatomy, retention of the articulated outer cuticle (Fig. 4). A ‘halo’ of sediment discolouration formed around the body of the specimens (unlike the trial with sediment but no specimen). In one buried specimen, the build up of decay gases served to form a bubble interior to the body. This acted as a locus for decay of the cuticle (i.e. formation of a hole in the body wall).

The loss of characters during decay is consistently non-random (i.e. interior non-cuticular characters vs exterior cuticular characters). Nevertheless, there is no evidence for synapomorphic decay bias (i.e. no correlation between decay rank of characters and synapomorphic rank, Spearman’s correlation coefficient p = 0.332). Cuticle characters do not demonstrate enough differential decay to test for synapomorphic decay bias within them, but it is highly unlikely given that oral scalids are extremely decay resistant and they yield both genus and phylum level characters (Figs 2 and 6).

The decay results from *Priapulus* enable identification of classes of characters that are unlikely to be preserved with fidelity under organic preservation i.e. non-cuticular anatomy and body proportions (given rapid taphonomic loss and alteration of body shape respectively). For the priapulid dataset^5^, 105 of a possible 812 entries (13%) are coded for the 29 parsimony informative non-cuticular/body proportion characters for the 28 organically preserved fossil taxa (compared to 70% for cuticular character entries of these taxa; Fig. 7). For the 26 extant taxa, 94% of non-cuticular character entries are coded and 86% of cuticular character entries are coded (Fig. 7). For the panarthropodan dataset^24^, 3% of entries for the 12 parsimony informative non-cuticular characters (15 entries) and 52% of entries for cuticular characters are coded for the 36 organically preserved fossil taxa (Fig. 7). For the 11 extant taxa, 87% of non-cuticular character entries are coded and 53% of cuticular character entries are recorded. Serially repeated mid-gut glands was not treated as a non-cuticular character given its consistent preservation through non-organic means (e.g. phosphatization^46^). Eliminating the non-cuticular character codings from organically preserved priapulid fossil taxa altered the topology of most parsimonious trees resulting from implied weighting searches (*k* = 3); a large group of fossil taxa, including *Ottoia* and *Louisella*, change affinity from that of stem-group priapulids to stem-group of the unnamed clade Kinorhyncha + Loricifera (Fig. 7). *Selkirkia* and *Paraselkirkia* also shift position from crown-group priapulids to stem-group scalidophorans. These character coding changes also reduce the amount of homoplasy as measured by the retention index (rises from 0.796 to 0.805). However, this change is found to fall within the range expected given random character coding elimination in the same proportion for each taxon (60 of 1000 random replicates had a retention index of 0.805 or higher giving p = 0.061). The resolution of the strict consensus tree is also improved following systematic character coding elimination (46 to 48 resolved nodes), but this again falls within the range expected given random character coding elimination in the same proportion (114 of 1000 random replicates recovered 48 or more nodes in the strict consensus tree, giving p = 0.115). Qualitatively similar results are obtained from searches with and without implied weighting, and searches with or without new tree search technology^45^,^47^. Applying the same tests to the panarthropodan dataset^24^ finds that the strict consensus tree topology does not change following systematic elimination of character codings for the organically preserved fossil taxa (Fig. 7). Again, homoplasy is reduced following this change, but only marginally (retention index from 0.8851 to 0.8853); once again this is within the ranges expected given random character coding elimination in the same proportion (p = 0.137 or 0.159 with and without implied weighting respectively).

Following the test for individual taxon slippage, 14 of the 25 extant taxa of the priapulid dataset shifted down the tree from their original position, toward the root, following elimination of non-cuticular character codings, compared with 9 that shifted up. Only one of those taxa, *Halicryptus spinulosus*, shifts significantly down the tree when compared to random character coding elimination in the same proportion. Both fall within the range expected by chance and are non-significant at p = 0.05. Of the 10 extant taxa of the panarthropodan dataset, 7 undergo no taxon shift at all following elimination of non-cuticular change codings. Of those three that do change position, two shift down, one shifts up. The later, *Hypsibius dujardini*, is also the only shift that is significant compared to random character coding elimination in the same proportion.

## Discussion

**Figure 1:**
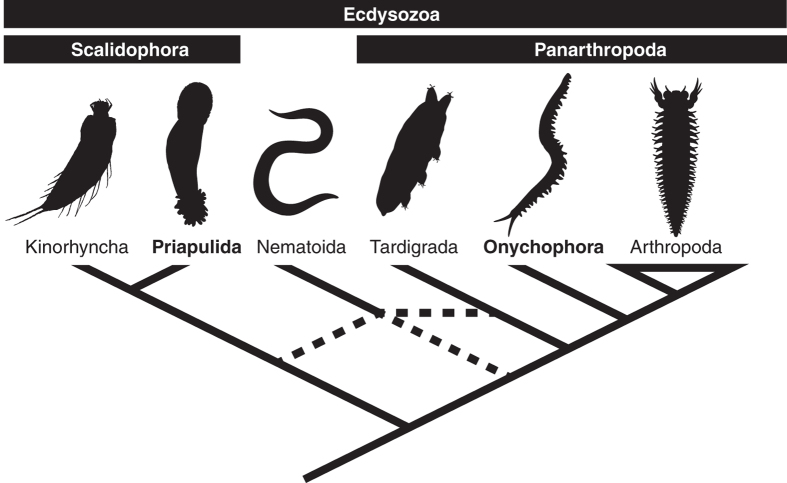
Phylogeny of major ecdysozoan clades 8,9,10,11,12. Images from PhyloPic: Kinorhyncha (“ http://phylopic.org/image/cd62afdf-5b96-44fc-89b7-60d018cd4d5a”) by Noah Schlottman, and Martin V. Sørensen, is licensed under the Attribution-ShareAlike 3.0 Unported license (“ http://creativecommons.org/licenses/by-sa/3.0/”), Arthropoda (“ http://phylopic.org/image/1353c901-f652-4563-941d-7b12bc7a86df”) by Gareth Monger is licensed under the Creative Commons Attribution 3.0 Unported license (“ http://creativecommons.org/licenses/by/3.0/”), Priapulida (http://phylopic.org/image/12ea611a-0911-4e3a-9eae-2fe0d46858e6”) and Onychophora (http://phylopic.org/image/7bb7abc6-1ab0-4116-a4eb-672f5f862386”) both available under public domain).

Experimental investigations of priapulid anatomical decay revealed two main consistent and predictable patterns. Firstly, there is a marked difference between the decay susceptibility of cuticular and non-cuticlar anatomy; the softer anatomy of the anterior (e.g. muscles, nerves) are lost extremely rapidly whilst the scalids and outer cuticle remain articulated, retaining fine anatomical detail well into the later stages of decay. Secondly, body shape undergoes extreme and rapid distortion during decay; within a period of 48 hours from death, specimens expand, contract, and bend, changing not only body size, but also the relative proportions of regions. These patterns are consistent for trials with and without sediment. Whilst we might reasonably expect the patterns from *Priapulus* to be general for the phylum Priapulida, to ascertain more broadly we need to apply the principle of comparative taphonomy^27^. Comparison with the decay of two genera of Onychophora^29^ finds the patterns of decay observed in priapulids to be consistent across these two phyla: interior anatomy decays rapidly and undergoes shrinkage following separation of the outer cuticle and the epidermis. Furthermore, the body expands, resulting in bloating and bending of the body and change of relative body region proportions; the pattern is therefore consistent for marine Priapulida and terrestrial Onychophora. Given this consistency across the two phyla (and a lack of data for related phyla), we can generalize these decay patterns for all early ecdysozoans (Fig. 1). This includes not only the clades closely related to Priapulida (i.e. the phyla Kinorhyncha and Loricifera) but also the clades phylogenetically intermediate to Scalidophora and Onychophora (i.e. the Tardigrada and Nematoida). Furthermore, the decay patterns should also apply to at least some of the arthropod stem, including, but not limited to, the arthropodan ‘lobopodians’.

Application of these decay patterns serves as test of models of preservation of early ecdysozoans. That cuticle, as a tissue, is generally resistant to decay is consistent with models of Burgess Shale-type preservation. The enhanced preservation potential of cuticle, and thus cuticle characters, is experimental demonstrated here for organic modes of preservation. Preservation of non-cuticular anatomy (e.g. muscles) is not inconsistent with these models and experimental data, as long as it is preserved through rapid modes e.g. phosphatization^48^. Current understanding of models of preservation and empirical patterns of decay indicate that preservation of unpigmented non-cuticular anatomy through organic preservation pathways is unlikely, at least with any degree of fidelity. Gaps between the internal tissue mass and the outer cuticle form rapidly as a result of either shrinkage of the former, expansion of the later, or both. As such, internal margins or lines in fossils^49^,^50^,^51^ could alternatively be interpreted as margins of a shrunken internal mass rather than representing discrete anatomy such as muscles or nerve cords. The rapid decay of non-cuticular anatomy also presents a direct challenge to recent interpretations of neural tissues preservation in fossil lobopodians^29^,^51^, and now more generally for all early ecdysozoan fossil taxa^21^,^22^,^23^,^24^,^25^,^26^,^28^. This does not necessarily rule out all fossil neural tissue interpretations, but it does mean that interpretations require further justification e.g. development of a new taphonomic model or new taphonomic data.

Application of these decay patterns also serves as a test of phylogenetic reconstruction in light of empirical taphonomic loss and alteration. Two classes of characters are identified as unlikely to be preserved with fidelity given classic organic preservation: unpigmented non-cuticular anatomy and body dimensions. These characters were found to be recorded infrequently for scalidophoran fossil taxa (13% of possible entries coded, of which nearly half related to body dimensions), and extremely rarely for panarthropodan fossil taxa (3% possible entries coded, no characters related to body dimensions). For both datasets, applying equivocal coding to non-cuticular characters in organically preserved fossil taxa gave more consistent results (reduced homoplasy), but this was not outside the range expected given random missing data in the same proportion. Nevertheless, non-cuticular character codings were found to play a pivotal role in reconstructing relationships of scalidophorans. A large group of fossil taxa are resolved as either stem-priapulids or on the stem of Loricifera + Kinorhynchs depending on whether the coding for the taphonomically unlikely character codings of fossil taxa are, or are not, included (Fig. 7). Furthermore, no fossil taxa at all are recovered on the priapulid stem without non-cuticular character codings. In the case of the panarthropodan dataset, the same topology is recovered under either non-cuticular character coding approach. So whilst the preservation and interpretation of neural tissue in early ecdysozoans has attracted a lot of attention recently^20^,^21^,^22^,^23^,^24^,^25^,^28^, it seems that the data yielded has yet to achieve much phylogenetic impact.

**Figure 2:**
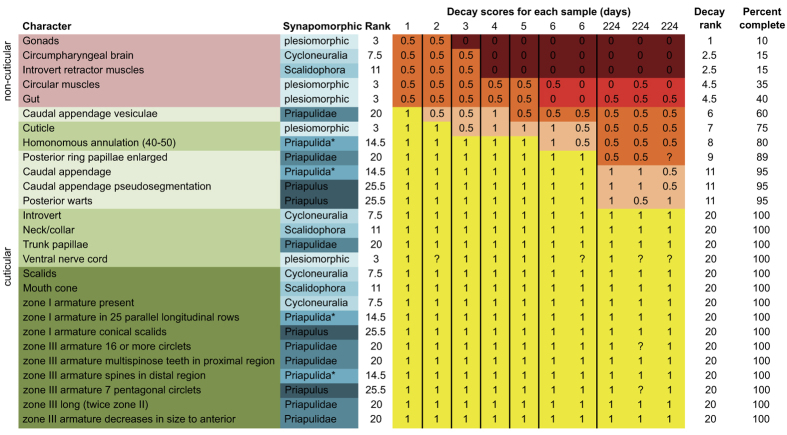
Characters of *Priapulus* and their decay profiles. Characters are classified as either cuticular (green: head region, trunk region or caudal region) or non-cuticular (pink) and by their synapomorphic rank (asterisks mark characters that are general for Priapulida but not observed in all members of the phylum). The ventral nerve cord is treated as a cuticular character because it is principally observed as a groove in the external cuticle surface. Characters are ranked according to decay profile (1 is pristine, 0.5 is exhibiting decay, 0 is lost) and colour coded following Sansom *et al*.^35^,^36^. Observations are scored as missing where that particular feature was unobservable in a specimen.

**Figure 3:**
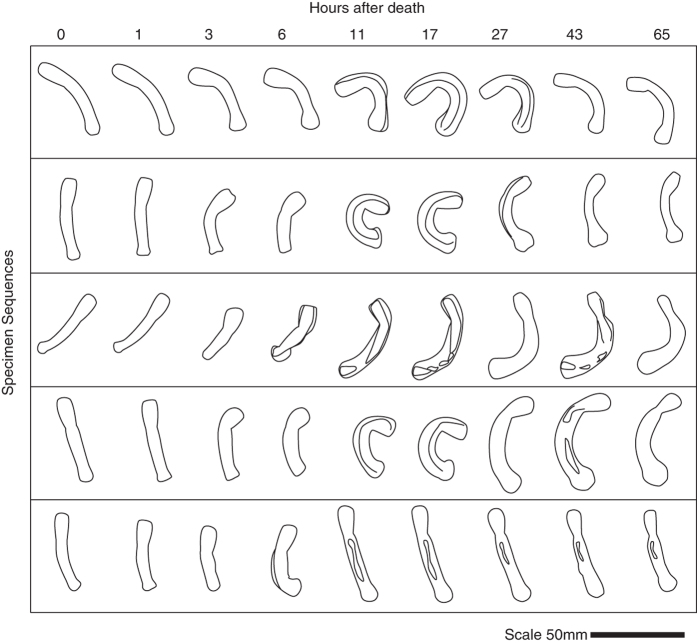
Distortions of external and internal body margins of *Priapulus.* 5 specimens decaying over 65 hours. See supplementary movies.

**Figure 4:**
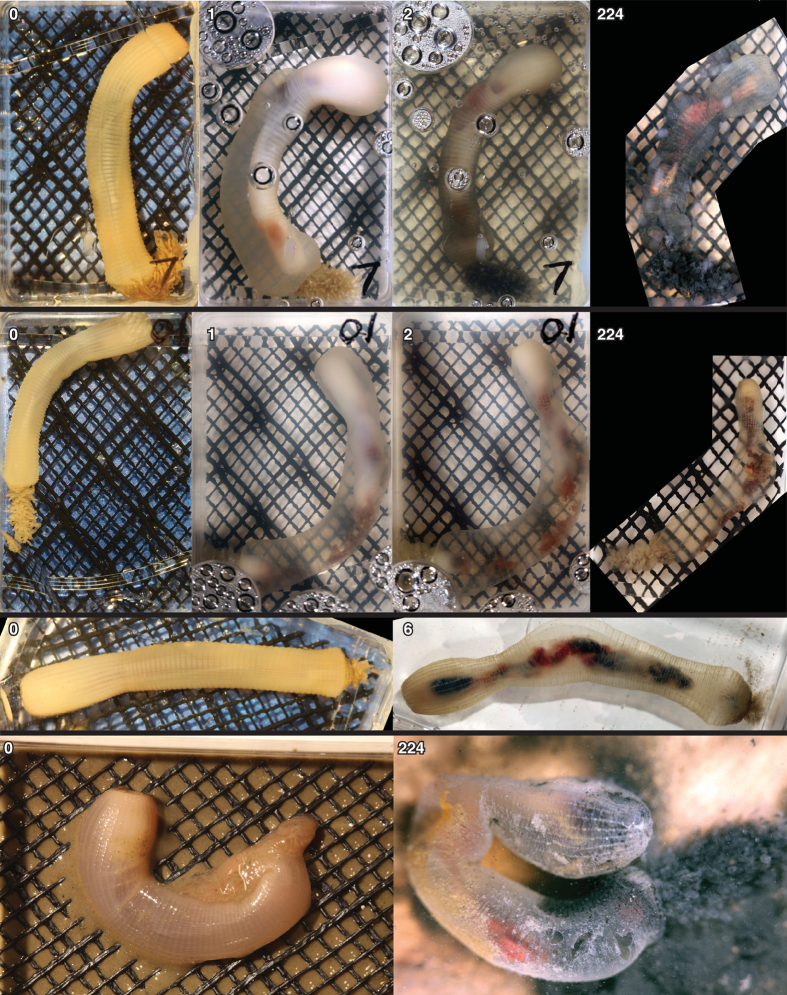
Gross morphological changes of *Priapulus*. Four specimens showing shrinking of internal anatomy (with number of days of decay). Mesh apertures approximately 2 mm × 2 mm. Bottom row for specimen encased in sediment (not to scale).

The data from priapulids revealed no evidence of any synapomorphic decay biases (Fig. 2). The same is true of decay data from Onychophora^29^. The currently available data therefore indicate that the absence of synapomorphic decay bias is general for early ecdysozoans and as such, there is no evidence for stem-ward slippage bias relating to preferential loss of synapomorphies during decay^35^,^36^. Application of the observed decay bias (loss of non-cuticular anatomy) to phylogenetic data also finds no evidence of significant stem-ward slippage. Removal of non-cuticular anatomy or body dimension character codings from extant taxa does not result in significant stem-ward slippage i.e. taxa do not undergo any systematic shifts in position following application of taphonomic filters.

**Figure 5:**
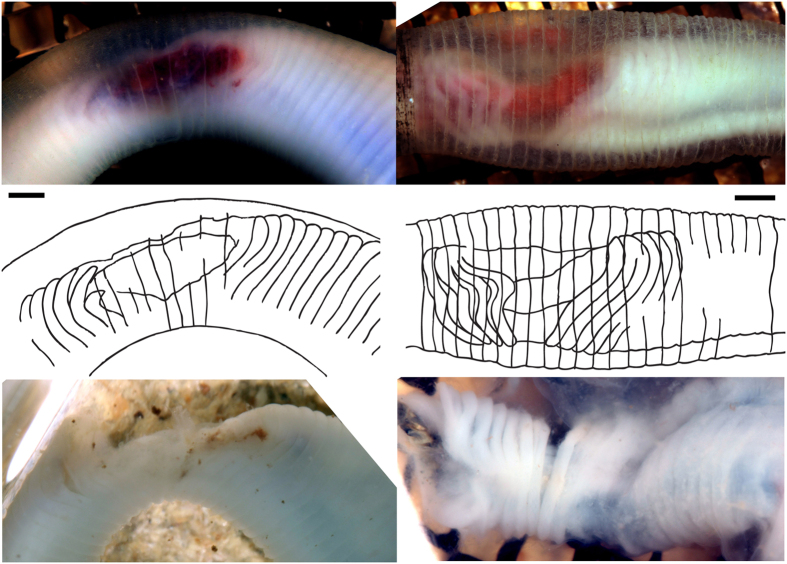
Decay of circular muscles in *Priapulus*. Change from annulations to sigmoidal shapes after 24 hours (left) and 48 hours (right), with external anatomy (top, levels uniformly adjusted to enhance muscle boundaries), and dissected muscles (below). The rings of cuticle are disjoined from the rings of circular muscles. Scale bars 2 mm.

**Figure 6:**
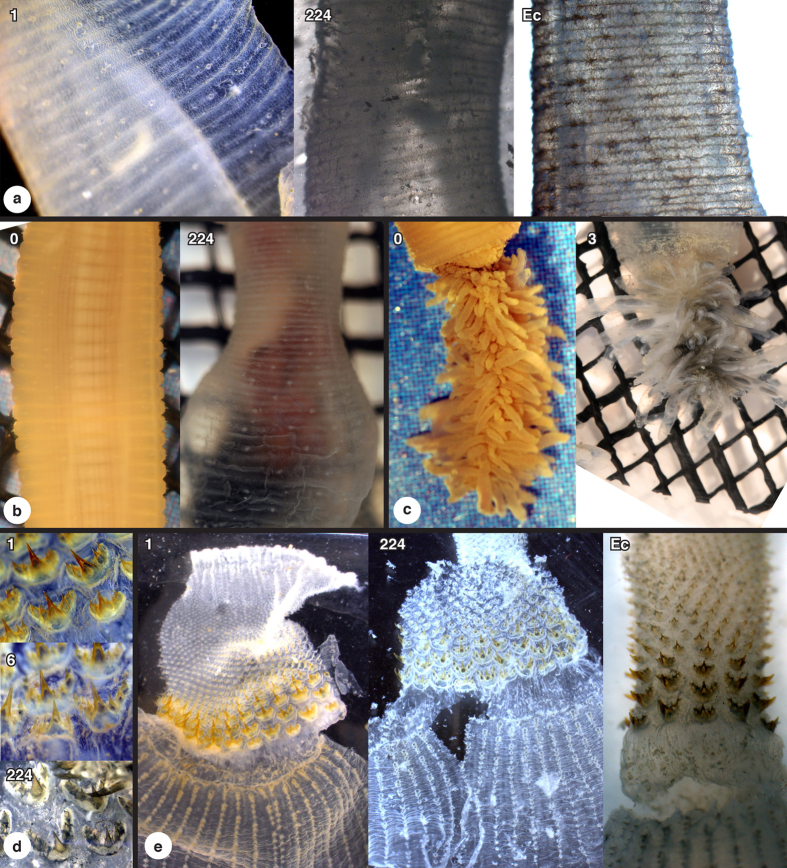
Decay of cuticular anatomy in *Priapulus*; (**a**) annulated trunk cuticle; (**b**) relative fraying and expansion of trunk cuticle; (**c**) caudal appendage with shrinkage of internal anatomy; (**d**) oral scalids details; (**e**) oral scalids and proboscis (number of days of decay, Ec is ecdysis moult). Mesh apertures approximately 2 mm × 2 mm.

**Figure 7:**
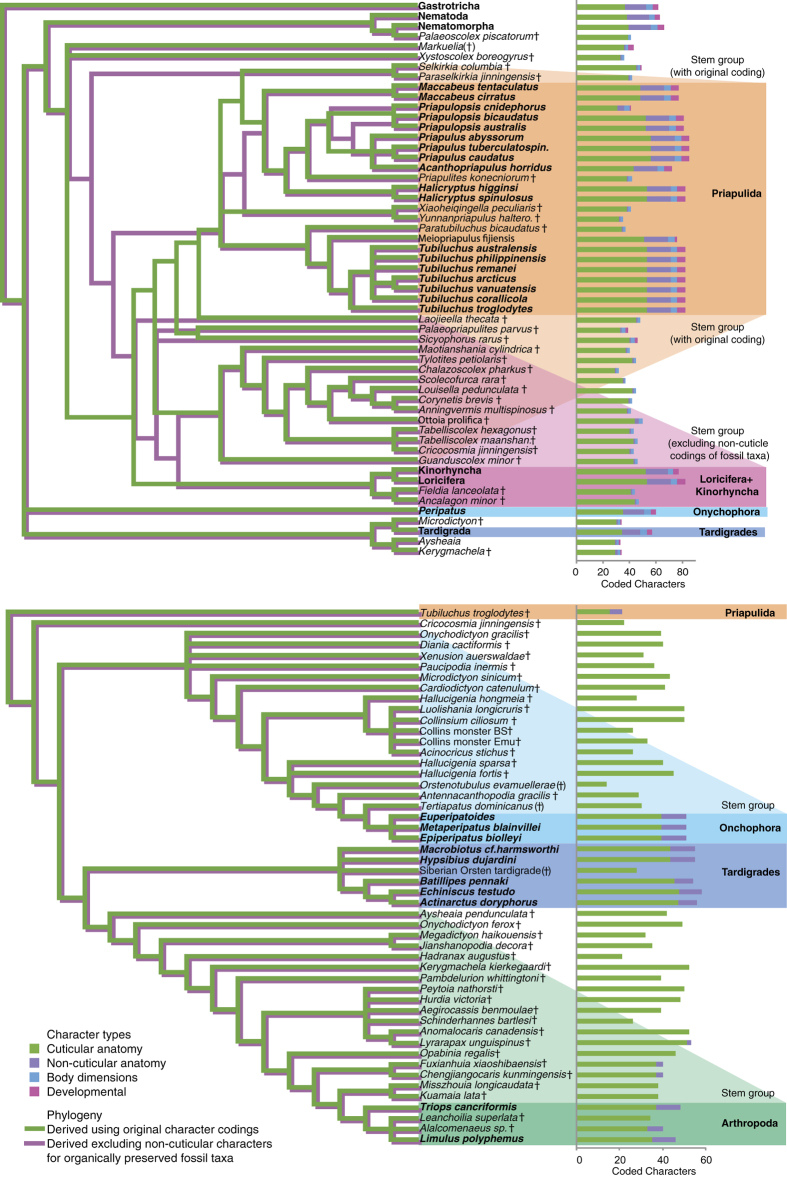
Phylogeny of Scalidophora^5^ (above) and Panathropoda^24^ (below). Original coding (green tree) and taphonomically informed coded (i.e. equivocal coding for non-cuticular characters of organically preserved fossils; purple tree). Taxa are either extant (bold) or extinct (dagger for organically preserved taxa, bracketed dagger for other fossil taxa). The numbers of parsimony informative character codings are given for each taxon (bar charts, right), broken down by character types.

Unexpectedly, the patterns of decay in *Priapulus* have relevance to interpretation and identification of fossil chordates. A key feature of chordates is segmental muscle blocks (myomeres) arranged in V or W-shapes. This distinguishes chordates from the vermiform bauplans such as those of priapulids or many annelids, in which homonomous annulation is observed i.e. rings of circular muscles corresponding with segments. Identification of angled segmental muscle blocks has become of central importance for diagnosis of fossil taxa as having chordate affinity. In some cases, putative muscle blocks are observed to exhibit marked sharp angle of inflection e.g. conodonts^46^, *Metaspriggina*^52^,^53^. In other cases, more subtle forms of curvature (sigmoidal, S-shapes) have been inferred as indicating chordate affinities e.g. *Pikaia*^19^, *Tullimonstrum*^54^. During the early stages of decay of *Priapulus* however (within 48 hours post death), the circular muscles can undergo transformation whereby the ring shape becomes sigmoidal or even double sigmoidal from a lateral perspective (Fig. 5). As such, taphonomic changes can create what has been inferred to be a chordate characteristic in a non-chordate organism. This potentially undermines the rationale that sigmoidally curved muscle segments indicate chordate affinity^19^,^54^,^55^. On the other hand, the taphonomic transformation and loss of V- and W-shaped myomeres of *Branchiostoma* and vertebrates respectively have been described^41^ and used as support for interpretation of curved structures as chordate muscle blocks^55^. Furthermore, the rapid decay of priapulid circular muscles makes preservation unlikely under organic preservation. Given the taphonomic ambiguity of subtly curved segments, it is appropriate for future analyses to focus on sharp angles of inflection (which is the condition observed in extant chordates) as providing support for chordate affinity.

## Conclusions

Cuticle, and by consequence cuticular characters, are demonstrated to be resistant to decay whilst non-cuticular anatomy and characters are quick to decay. These experimental results are therefore consistent with current understanding of Burgess Shale-type preservation. Following those models and the decay data, non-cuticular anatomy is unlikely to be organically preserved with fidelity (although could be preserved via authigenic mineralization, or if pigmented). Given the consistency of this pattern across priapulids and onychophorans, as well as decay related changes in body size, we can infer that those taphonomic patterns are general for at least non-arthropodan ecdysozoans and their Cambrian fossil record (i.e. scalidophorans, nematoids, tardigrades, and onychophorans). Although we lack similar data for arthropods, this taphonomic condition is resolved as plesiomorphic for the group and will apply to at least part of the arthropod stem (including those that are lobopodians). Applying taphonomic patterns to phylogenetic data finds that the taphonomically improbable character codings of organically preserved fossils play and important role in reconstructions of relationships of scalidophorans (including priapulids), but not panarthropodans.

Results presented here indicate that close scrutiny should be applied to taphonomic and anatomical interpretations of non-cuticular anatomy in organically preserved soft-bodied ecdysozoans. This includes the recent high-profile interpretations of neural and vascular tissues in Burgess Shale-type preserved ecdysozoans^20^,^21^,^22^,^23^,^24^,^25^,^26^. An inconsistency is identified between those anatomical interpretations and current understanding of preservation. As such, interpretations of neural and vascular tissue structures may require revisiting, or alternatively, formulation of new taphonomic models to account for their organic preservation is required.

## Additional Information

**How to cite this article**: Sansom, R. S. Preservation and phylogeny of Cambrian ecdysozoans tested by experimental decay of *Priapulus*. *Sci. Rep.* **6**, 32817; doi: 10.1038/srep32817 (2016).

## Supplementary Material

Supplementary Video 1

Supplementary Video 2

Supplementary Information
