# Supplementary material for: Preservation and phylogeny of Cambrian ecdysozoans tested by experimental decay of Priapulus
Source: Sci Rep. 2016 Sep 6;6:32817. doi: 10.1038/srep32817 (PMC5011709; doi:10.1038/srep32817)
Supplement: Supplementary Information [file srep32817-s3.doc]

**Supplementary Information for**

**Preservation and phylogeny of Cambrian ecdysozoans tested by experimental decay of *Priapulus***

*Robert Sansom*

School of Earth and Environmental Sciences, University of Manchester, Manchester M13 9PT, UK. robert.sansom@manchester.ac.uk

**Supplementary videos 1 and 2**

During the first 6 days of decay, time-lapse photography was used to record the state of specimens of *Priapulus* whilst in their containers. The photographs (1 per hour) were used to produce videos (supplementary videos 1 and 2, created using *Adobe Photoshop*). The videos demonstrate the gross morphological changes that took place during the short period of time, specifically dramatic changes in body proportions and the shrinkage of internal anatomy from the external cuticle.
